# Don’t Lay Your Eggs All in One Basket: Brood Parasitism as a Survival Strategy

**DOI:** 10.3201/eid2110.AC2110

**Published:** 2015-10

**Authors:** Byron Breedlove, Paul M. Arguin

**Affiliations:** Centers for Disease Control and Prevention, Atlanta, Georgia, USA

**Keywords:** art science connection, emerging infectious diseases, brood parasitism, John James Audubon, Cow-pen Bird, Plate XCIX (99), don’t lay your eggs all in one basket: brood parasitism as a survival strategy, parasites, parasitic diseases, protozoa, helminths, ectoparasites, art and medicine, about the cover

**Figure:**
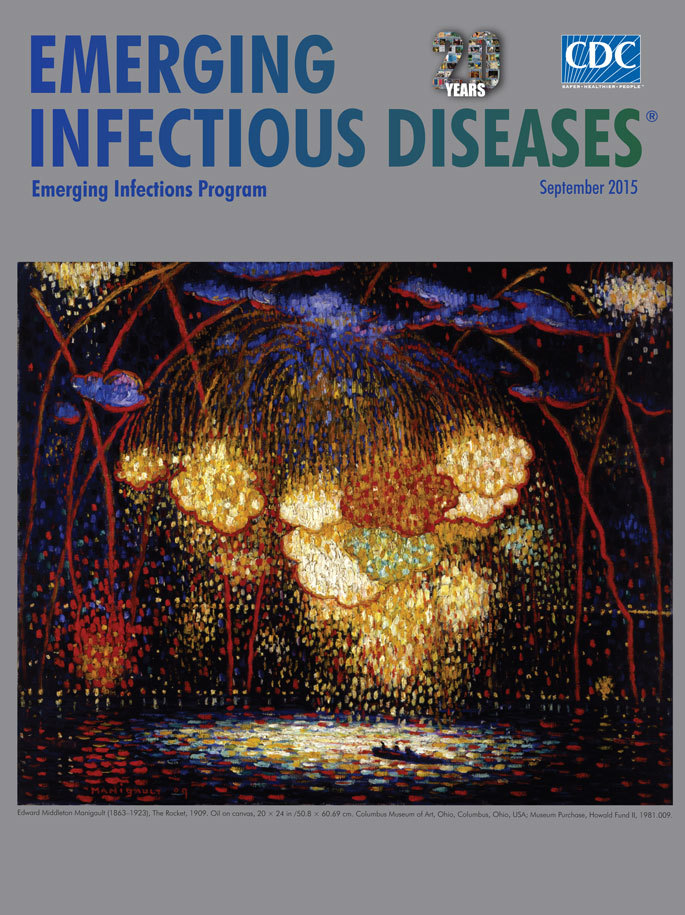
**John James Audubon (1785–1851). Cow-pen Bird, Plate XCIX (99) Engraved from watercolor. 19.5 × 12.25 in/49.53 × 31.12 cm.** Images from Archives Service Center, University of Pittsburgh, Pittsburgh, PA, USA

This month’s cover image,^1^ Plate 99 from *Birds of America* (printed in stages during 1827−1838) by American ornithologist, naturalist, and painter John James Audubon (1785–1851), shows a pair of oft-vilified brown-headed cowbirds. This painting appears in the book as one of 435 life-sized watercolors that were reproduced from Audubon’s hand-engraved plates. In this painting, he portrays the birds frozen in act of foraging, a technique he honed from observing birds where they lived. Audubon’s own words best describe the work: “Male with the head and neck sooty-brown, the body black, glossed with green, the fore part of the back with blue. Female considerably smaller, greyish-brown, the lower parts lighter.”

The genus of this bird, *Molothrus ater*—as well as the bronze-headed variant *Molothrus aeneus—*comes from Molothrus, the Greek work meaning vagabond or parasite. Although there are other bird species that prey on the parental skills of their feathered neighbors by laying eggs in their nests, only birds of those two species practice obligate parasitism in North America, placing them among approximately one percent of avian species worldwide.

Before cowbirds—which are also variously known as cow-pen birds, cow buntings, or buffalo birds—followed cattle, they tracked bison herds across the Great Plains, where they were sustained by the copious insects. Naturalists have ventured that cowbirds adapted to this nomadic existence by becoming brood parasites and depositing their eggs in nests built and incubated by birds of other species. Incubation for birds is analogous to pregnancy for mammals, providing warmth, protection, and food to the developing embryo. This process can involve establishing and defending a territory, nest building, and collecting food not for individual consumption, but for feeding the newly hatched chicks.

David Attenborough in his book *The Life of Birds* succinctly describes the advantages of such an adaptation: “Brood parasitism relieves the parasitic parent from the investment of rearing young or building nests for the young, enabling them to spend more time on other activities such as foraging and producing offspring. The risk of egg loss is mitigated, as eggs are distributed amongst a number of different hosts.” During a laying season, brood parasites often produce many more eggs than their parasitized hosts. By distributing these eggs among many different nests, they also spread the risk for nest predation, increasing the chances that some of their offspring will survive.

Skillful at stealth and stalking, brown-headed cowbirds parasitize birds of about 200 other species throughout North America; researchers have observed that birds of 144 of those species have raised cowbird offspring. Raising cowbird hatchlings among a brood extracts a toll on host species, despite countermeasures ranging from rejecting or destroying the intruder’s eggs or chicks to abandoning the parasitized nests. Cowbird eggs usually hatch a day ahead of the host’s own eggs, and cowbird nestlings usually are larger and mature faster than the host’s young, enabling them to consume more of the food their foster parents bring to the nest. Despite the fact that 97% of cowbird eggs and nestlings do not survive to adulthood, brood parasitism by cowbirds has pushed birds of some host species to the status of “endangered” and has probably hurt populations of birds of some other host species.

Brood parasitism, of course, is but one variant on the nonsymbiotic relationship between species, both animals and plants, in which one benefits at the expense of the other. Medical parasites are often larger and more complex than the average bacterium or virus, yet they depend on other organisms to complete their life cycle. They live on or in other species, using them as a food source, for shelter, or for some other essential aspect of their daily life. The actual number of parasitic organisms on earth is likely inestimable and certainly debatable; some researchers have postulated that 40% of the species in any location are parasitic.

Three main classes of parasites—protozoa, helminths, and ectoparasites—cause numerous diseases afflicting millions of humans. Of all parasitic diseases, malaria causes the most deaths globally, and other parasitic diseases such as lymphatic filariasis, onchocerciasis, and schistosomiasis afflict millions of people in the tropics and subtropics. Parasitic diseases can be imported into or in some cases transmitted within industrialized countries. In the United States, for example, tickborne transmission of babesiosis and foodborne outbreaks of cyclosporiasis and anisakiasis are just some of the parasitic infections of public health concern. Enhanced surveillance, treatment, and prevention of parasitic infections remain global health priorities.

## References

[1] Attenborough D. The life of birds. Princeton (NJ): Princeton University Press; 1998. p. 246.

[2] Audubon JJ. Birds of America. Plate 99: cow-pen bird [cited 2015 Aug 8]. https://www.audubon.org/birds-of-america/cow-pen-bird

[3] Centers for Disease Control and Prevention. Neglected parasitic infections (NPIs) in the United States [cited 2015 Aug 10]. http://www.cdc.gov/parasites/npi/index.html

[4] Dobson A, Laffert KD, Kuris AM, Hechinger RF, Jetz W. Homage to Linnaeus: How many parasites? How many hosts? Proc Natl Acad Sci U S A. 2008;105:11482–9 and. 10.1073/pnas.0803232105 18695218 PMC2556407

[5] Cornell Laboratory of Ornithology. Birds in forested landscapes. Brood parasite: brown-headed cowbird (*Molothrus ater*) [cited 2015 Aug 8]. http://www.birds.cornell.edu/bfl/speciesaccts/bnhcow.html

[6] Ehrlich P, Dobkin DS, Wheye D. Birder’s handbook. New York: Simon and Schuster; 1988. p. 289.

